# Diet-induced gut dysbiosis and inflammation: Key drivers of obesity-driven NASH

**DOI:** 10.1016/j.isci.2022.105905

**Published:** 2022-12-30

**Authors:** Gideon G. Kang, Natalie L. Trevaskis, Andrew J. Murphy, Mark A. Febbraio

**Affiliations:** 1 Monash Institute of Pharmaceutical Sciences, Monash University, Melbourne, VIC, Australia; 2 Baker Heart & Diabetes Institute, Melbourne, VIC, Australia

**Keywords:** Hepatology, Biological sciences, Physiology, Human metabolism, Immunology

## Abstract

Sucrose, the primary circulating sugar in plants, contains equal amounts of fructose and glucose. The latter is the predominant circulating sugar in animals and thus the primary fuel source for various tissue and cell types in the body. Chronic excessive energy intake has, however, emerged as a major driver of obesity and associated pathologies including nonalcoholic fatty liver diseases (NAFLD) and the more severe nonalcoholic steatohepatitis (NASH). Consumption of a high-caloric, western-style diet induces gut dysbiosis and inflammation resulting in leaky gut. Translocation of gut-derived bacterial content promotes hepatic inflammation and ER stress, and when either or both of these are combined with steatosis, it can cause NASH. Here, we review the metabolic links between diet-induced changes in the gut and NASH. Furthermore, therapeutic interventions for the treatment of obesity and liver metabolic diseases are also discussed with a focus on restoring the gut-liver axis.

## Graphical abstract

**Figure:**
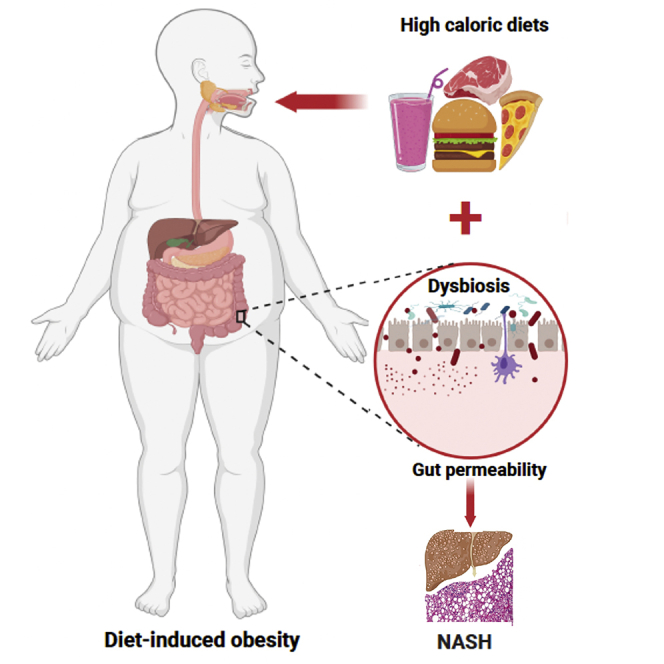

## Introduction

Macronutrients (including fats, proteins, and carbohydrates [CHO]) from food are absorbed across the intestinal wall following digestion and metabolic processing in the gut and from there are transported to distal organs where they are used as chemical energy for organ function.^1^ Complex CHO are digested to monosaccharides, including fructose and glucose, that are absorbed via glucose transporter (Glut) 5 and Glut2, also known as sodium-glucose-linked transporter-1 (SGLT-1).^2^^,^^3^ From there the monosaccharides are transported away from the intestine and directly to the liver via the mesenteric blood vessels and portal vein. Whereas proteins are digested to peptides and then to amino acids (AAs) which are transported to the liver via the portal vein where the toxic ammonia is rapidly converted to urea, which is eventually excreted in the urine.^4^ Dietary fats include triglycerides, phospholipids, and cholesterol derivatives. The main dietary fat, triglyceride, is sequentially digested to monoglycerides and fatty acids which are absorbed, resynthesized to triglyceride, and incorporated into lipoproteins. Unlike sugars, dietary fats are mainly transported from the intestine via the lymphatics as lipoproteins are too large to cross the blood vessel wall.^5^^,^^6^

The body stores surplus energy as fat in the adipocytes. However, chronic consumption of sugar-sweetened beverages (SSBs) and a high-fat diet (HFD) produces sustained positive energy balance where energy intake exceeds energy expenditure.^7^ Excessive positive energy balance promotes the deposition of fat in cells other than adipocytes, leading to metabolic perturbations and obesity.^8^ Obesity is usually characterized by an increased body mass index of ≥30 kg/m,^2,^^9^ and this has been associated with a higher overall mortality rate in both males and females.^10^ Thus, diet-induced obesity (DIO) has emerged as the major risk factor for the development of many chronic conditions including type 2 diabetes mellitus (T2DM), cardiovascular diseases (CVD), some types of cancers such as breast, liver, and colon cancers, and liver diseases (e.g., nonalcoholic fatty liver disease [NAFLD] and nonalcoholic steatohepatitis [NASH]). NAFLD ranges from benign steatosis, characterized by moderately increased triglyceride content in hepatocytes, to NASH where there is additional inflammation present, and more severe liver cirrhosis with fibrosis.^11^ The progression from NAFLD to NASH involves multiple insults including inflammation and cellular stress, such as oxidative and ER stress. This phenomenon is referred to as the multiple hit hypothesis.^12^^,^^13^ Although it is not the scope of the current review, host genetics also play a key role in modulating the development of chronic diseases, as reviewed by Ussar, Fujisaka, and Kahn.^14^ Consumption of SSBs and HFD drives the development of obesity, a major risk factor for NAFLD in developed countries.^15^^,^^16^ The link between obesity and NAFLD/NASH has been appreciated in bariatric surgery studies where NAFLD was detected in 85%–95% of patients with obesity,^17^ aligning with a meta-analysis report in which patients with NAFLD (51%) and NASH (82%) were found to be obese.^18^ DIO promotes the accumulation of lipid droplets in the liver parenchyma leading to the development of NAFLD.^19^

**Figure 1:**
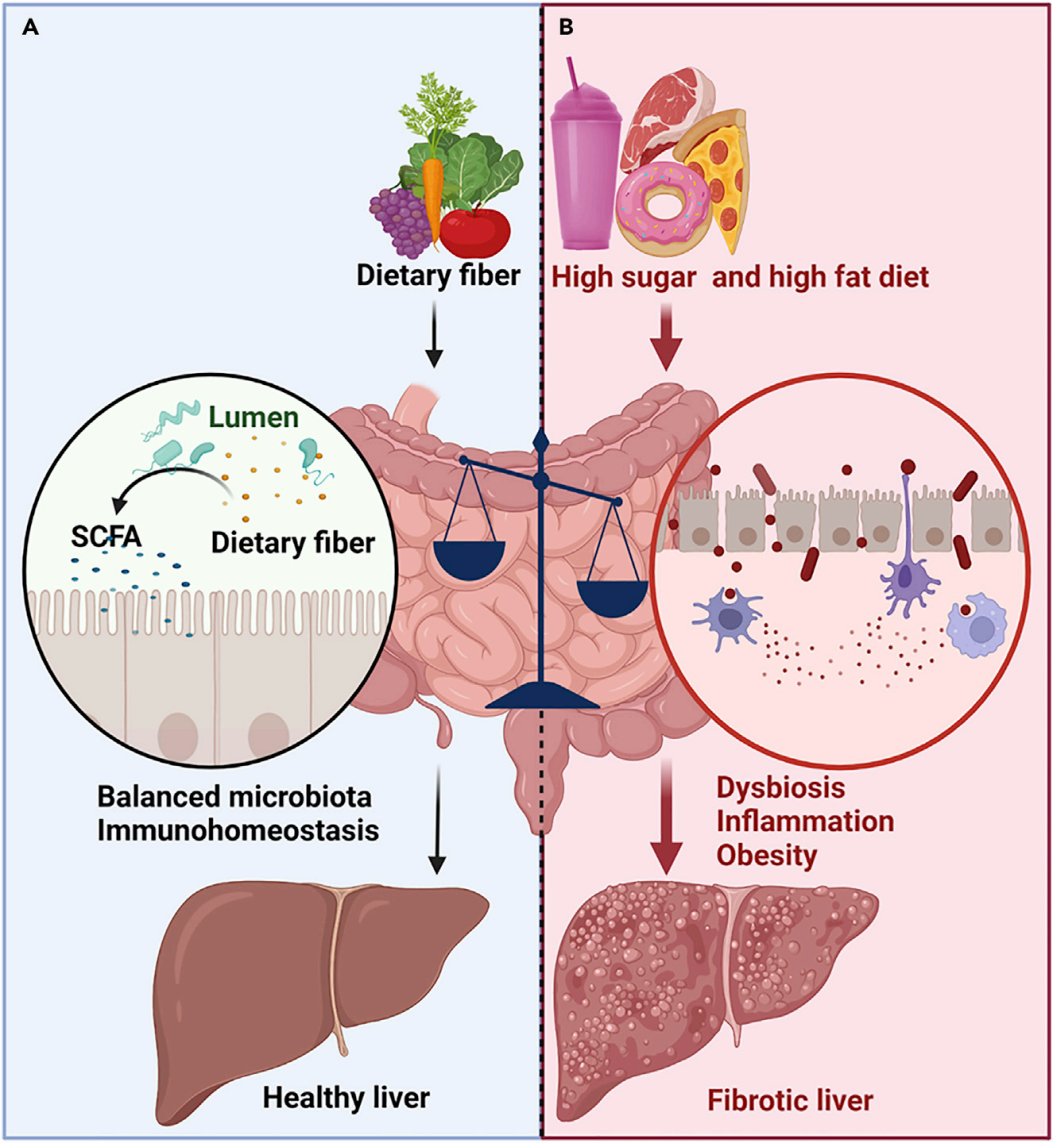
The effect of macronutrients on the gut and liver (A) normal nutrient metabolism. In health, dietary fiber is metabolized by gut bacteria to produce short-chain fatty acids (SCFAs), which maintain gut barrier function. This protects the liver from gut-derived endotoxins. (B) altered nutrient metabolism. Dietary imbalance induces gut dysbiosis and increases gut permeability. This increases the translocation of gut content to the liver, promoting the development of nonalcholic fatty liver diseases (NAFLD) and nonalcholic steatohepatitis (NASH). Arrows denote sequential metabolic effects. Broader arrows indicate greater effect. The balance scale indicates the imbalance in dietary consumption. This figure was created with Biorender.com.

The mechanisms of diet-induced NAFLD and NASH are complex. However, gut microbiota (the assemblage of microorganisms in the gut) has been shown to play a pivotal role in gut energy metabolism.^20^ Highly processed western-style diets (WD) containing high sugar, high fat, and high cholesterol are rapidly absorbed in the small intestine with a very small amount reaching the colon. This deprives the gut microbiota the essential nutrients needed for bacterial growth, promoting gut dysbiosis—an imbalance in the number and/or diversity of the microbiota; whereas, consumption of fiber-rich plant-based diet is typically considered to promote gut eubiosis,^21^ a normal physiological state where a balanced microbial abundance and diversity is maintained^22^ (Figure 1). Gut dysbiosis is usually described based on the Firmicutes/Bacteroidetes ratio, an increase of which is a marker of altered microbiota-induced obesity,^23^ albeit this remains controversial. WD-induced gut dysbiosis favors an increase in inflammation-evoking gut bacteria leading to inflammation and enterocyte ER stress. Emerging evidence has demonstrated that consumption of a high-fructose diet (HFrD) downregulates tight junction proteins (TJPs) and concomitantly increases hepatic *de novo* lipogenesis (DNL)—the production of new lipids from glucose and fructose by activation of the enzyme acetyl-coenzyme A (CoA) carboxylase (ACC).^24^^,^^25^ The downregulation of TJPs, by HFrD, promotes gut barrier deterioration resulting in the leakage of gut-derived metabolites and bacterial products such as lipopolysaccharides (LPSs, glycolipids found on the cell wall of gram-negative bacteria) into the portal circulation reaching the liver. In the liver, LPS binds to Toll-like receptor 4 (TLR4) on the macrophages and activates the release of host-derived inflammatory mediators triggering liver inflammation and the development of NAFLD/NASH.^24^

Although the literature has advanced our understanding of the effects of high-caloric diets on gut and liver pathologies, it remains unclear whether specific macronutrients have distinct effects. It’s well understood that sugar consumption *per se* does not lead to obesity when a diet is isocaloric. In well-controlled human studies, a fructose-rich diet does not increase body weight when compared with a diet matched for energy from glucose.^26^ Nevertheless, fructose elicits more deleterious liver pathology than glucose; highlighting the complex interplay between diet and obesity-induced NAFLD/NASH. Such complexity, in part, hinders scientific progress for a suitable therapeutic treatment for NASH. The list of drugs for obesity treatment is extensive, but there is no suitable drug treatment for NASH, leaving lifestyle modification through diets and exercise and bariatric surgery as the only available therapeutic options.^27^ Herein, we review the current knowledge on the biology of macronutrients and their modulation of gut metabolism and how this influences the development of liver diseases via the gut-liver axis. We also discuss the potential of some new therapeutic targets to treat obesity-related diseases and their effects on gut-liver energy shuttling.

## Nutrient absorption and metabolism

### Carbohydrates: Fructose and glucose

Although sucrose, the primary circulating sugar in plants, contains equal amounts of fructose and glucose, glucose is the predominant circulating sugar in animals and thus the primary ubiquitous fuel source for both anaerobic and aerobic cellular metabolism in various body tissues. Structurally, glucose and fructose are both hexoses containing 6 carbon atoms in the same chemical formula C_6_H_12_O_6_. This encouraged the early views that glucose and fructose are equivalent and, therefore, must have indistinguishable physiological effects.^2^ However, the functional group positioning (where an aldehyde is attached to carbon 1 of glucose, and keto is attached to carbon 2 of fructose) presents a critical variation of the two structures, with glucose forming a 6-membered ring and fructose, a 5-membered ring. Whether these differences could potentially influence biological parameters such as digestion, absorption, and metabolism is unclear.

Fructose is absorbed from the intestine into the enterocytes via GLUT5,^28^ possibly owing to higher specificity of Glut5 for fructose over glucose although with a ranging Km (6–15 mM) for fructose.^29^^,^^30^ The expression of Glut5, high at the apical pole (AP) but less on the basolateral pole (BLP), is influenced by dietary fructose ingestion.^29^ Dietary fructose-induced expression of Glut5 is linked to the activity of carbohydrate-responsive element-binding protein (ChREBP), often referred to as Mlxipl. Accordingly, Barone, Fussell, Singh, Lucas, Xu, Kim, Wu, Yu, Amlal, Seidler, Zuo and Soleimani,^31^ and the others^32^^,^^33^ have shown that deletion of ChREBP reduced both intestinal fructose absorption and serum fructose concentration by more than 80%, resulting in severe features (including diarrhea and intestinal distension) of morbid malabsorption syndrome in mice. These data highlight the crucial contribution of Glut5 and ChREBP in fructose absorption.

**Figure 2:**
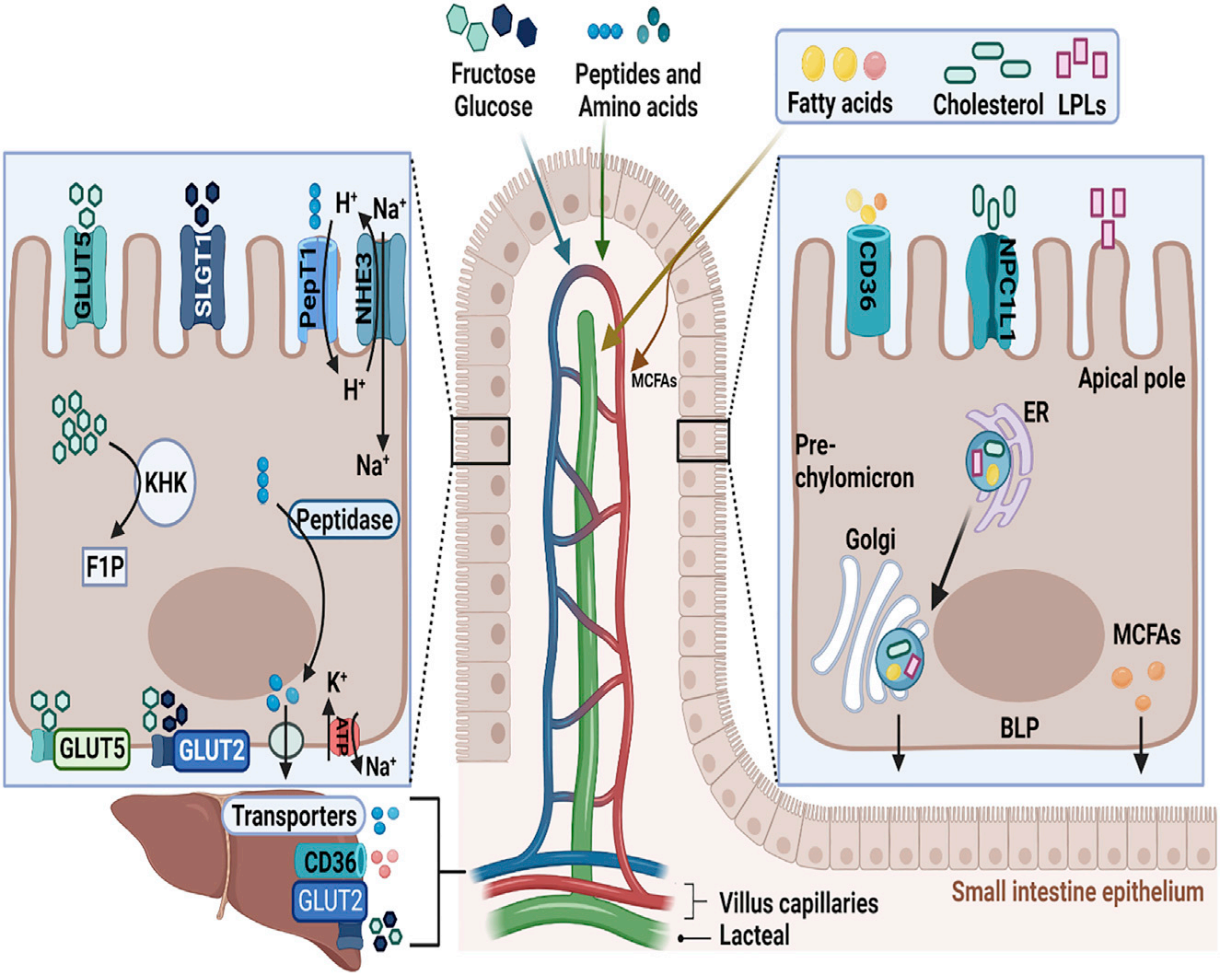
Macronutrient absorption and metabolism in the enterocyte and the liver At the apical pole of the enterocyte, fructose and glucose are absorbed via glucose transporter 5 (GLUT5) and sodium-glucose co-transporter 1 (SLGT1), respectively. The majority of the absorbed fructose is converted to fructose-1-phosphate (F1P) by ketohexokinase (KHK). The unmetabolized portion of fructose is transported across the basolateral pole (BLP) via GLUT5 to the portal vein. A smaller amount of fructose is also transported across the BLP along with glucose via GLUT2. Both fructose and glucose enter the liver through GLUT2. The absorption of di- and tripeptides is assisted by PepT1, facilitated by sodium/H+ exchanger 3 (NHE3). Amino acids are transported into the enterocyte via various transporters. Some amino acids are actively taken up through NHE aided by exchange of Na+. Peptides are cleaved by peptidases into AAs which passively diffuse into the portal circulation and enter the liver via various transporters. Sodium and potassium ions move out and into the enterocytes via Na+/K + -ATPase channel, the source of cellular energy. Fatty acids and mono-aclyglycerols formed from the digestion of dietary triacylglycerol are transported into the enterocytes either passively or facilitated by transporters such as CD36. Cholesterol is absorbed via Niemann-Pick C1-like 1 (NPC1L1), whereas lysophospholipids (LPLs) passively diffuse into the enterocyte. Long-chain free fatty acids, monoglycerides, cholesterol, and LPLs are shuttled to the ER where the fatty acids and monoglycerides are re-esterified to TAGs and cholesterol and LPL esterified to cholesterol esters and PL, respectively. These are packaged into pre-chylomicrons before transporting to the Golgi apparatus for maturation. Chylomicrons are exocytosed from the enterocyte and directly transported into the lymphatic system via lacteals. Medium chain fatty acids (MCFAs) are transported from the intestine via the mesenteric blood vessels and portal vein and from here directly enter the liver and hepatocytes via transporters such as CD36. This figure was created with Biorender.com.

Recent studies suggest that the majority (90%) of absorbed fructose is metabolized in the enterocytes, initiated by phosphorylation of fructose by ketohexokinase (KHK, also known as fructokinase), and only a small amount reaches the liver.^34^ This finding contrasts the previous views recognizing liver as the primary fructose-metabolizing organ and that only 10%–30% of the ingested fructose is metabolized in the small intestine.^35^^,^^36^ The KHK-C isoform, which has higher affinity for fructose than KHK-A,^37^ converts fructose to fructose-1-phosphate (F1P), a potentially toxic intermediate (Figure 2). Subsequent molecular reactions lead to the generation of glucose and fructose-derived metabolites including alanine, lactate, glycerate, and other organic acids, which are then funneled via GLUT2, at basolateral membrane of enterocytes, to the portal vein.^34^ In intestinal-specific KHK-C knockout mice, more than double the amount of fructose and significantly more gut-derived short-chain fatty acids (SCFAs, discussed subsequently) reached the liver via the portal vein; collectively inducing hepatic lipogenesis.^38^ These findings portray the small intestine as a major site for dietary fructose metabolism,^34^ which may provide protection against fructose spillover to the liver and colon and thus may shield the liver from excessive fructose exposure.^38^

The unmetabolized portion of fructose is transported across the BLP into the portal vein. How fructose crosses the BLP, however, remains the subject of debate. A prominent proposition that GLUT2 is the main transporter for both fructose and glucose^3^^,^^39^ has constantly been challenged by the finding that Glut2 has lower affinity (Km = 11 mM) for fructose compared to GLUT5 (Km = 6 mM),^40^ thus suggesting that GLUT2 is a minor contributor to fructose export. However, GLUT5 is not well expressed at the intestinal BLP,^29^ again supporting the widely accepted view that GLUT2 is the primary mediator for fructose transport.^3^^,^^41^ In the liver, fructose undergoes a similar metabolic fate as in the enterocyte, except that KHK has a high activity V_max_ (about 3 mmol/min/g wet weight) in rat liver, coupled with its higher affinity for fructose than glucose.^42^ Therefore, the majority of fructose that enters the liver via the portal vein is rapidly cleared before reaching the systemic circulation.^43^ Here, subsequent fructose catabolism leads to the production of citrate, which is further converted to acetyl-CoA by ATP citrate lyase (ACLY). Catalyzed by ACC1, acetyl-CoA is converted to malonyl CoA, which is fed into fatty acid synthesis by fatty acid synthase (FASN), producing C:16 and C:18 fatty acids. These are then synthesized into triacylglyceride (TAG) via conjugation with glycerol-3 phosphate (G3P), leading to the formation of lipid droplets in the liver.^2^

Contrary to fructose, glucose is actively absorbed by the enterocytes via GLUT2.^44^ GLUT2 is expressed in various tissues in the body including the liver, kidney, and the intestines and was initially identified as a transporter of both fructose and glucose into the enterocyte.^41^ This is possibly due to the fact that GLUT2 is expressed both at the luminal and BLP of the epithelial cells. Its ability to efficiently pump glucose against a cellular concentration gradient ensures a complete and fast absorption rate.^45^ Like fructose, however, glucose’s active transportation into the enterocytes can be saturated under a high intestinal glucose concentration after a meal, and as such, facilitated diffusion and paracellular transport are considered alternative routes.^46^ Upon entering the enterocytes, glucose is transported across the BLP through GLUT2 into the portal circulation, bypasses the liver clearance metabolism, and enters the systemic circulation where it’s transported to other metabolic tissues and used as fuel.

### Lipids

Dietary fat is predominantly composed of nonpolar lipids, including di- and tri-acylglycerols (DAGs and TAGs) and cholesterol esters, and polar phospholipids. Metabolites of dietary fats are vital energy sources, and associated lipid soluble vitamins are essential nutrients. The major dietary fat is TAG. Following their ingestion, dietary TAG is partially hydrolyzed in the stomach, by gastric lipase, into fatty acids and DAGs to facilitate formation of a crude emulsion. Upon entry into the small intestine, the action of pancreatic lipases further hydrolyzes TAGs and DAGs to monacylglycerol (MAGs) and fatty acids. Furthermore, mixing with bile components including bile acids and endogenous lipids (phospholipid and cholesterol) leads to the formation of highly solubilized lipid colloidal species from which lipids are taken up into enterocytes.^6^

Dietary-derived fatty acids and MAGs are taken up by the enterocytes either via passive diffusion due to the concentration gradient or active transport by transporters such as CD36 and fatty acid transport protein 4 (FATP4).^47^ A recent work in mice has shown that specific deletion of CD36 in lymphatic endothelial cells reduced lipid transport and promotes obesity and leaky gut lymphatics.^48^ Consistent with previous data, inactivation of CD36 and FATP4 reduces fatty acid uptake by 50% in primary mouse enterocytes^49^ and results in defective chylomicron assembly and secretion, highlighting the pivotal role of chylomicrons in lipid absorption (Drover, Ajmal, Nassir, Davidson, Nauli, Sahoo, Tso, and Abumrad).^50^

Cholesterol influx into the enterocytes, and efflux back into the lumen, are mediated by Niemann-Pick C1-like 1 (NPC1L1) and ATP-binding cassette subfamily G 5 and subfamily G 8 (ABCG5 and ABCG8). In contrast, lysophospholipids absorption is believed to be facilitated by passive diffusion through the AP.^49^^,^^51^ In the enterocyte, absorbed fatty acids and MAGs are resynthesized to TAGs at the smooth ER and then either packaged into chylomicrons or stored as cytosolic lipid droplets (CLDs).^52^^,^^53^ Pre-chylomicrons are formed as TAG budding from the interior of the smooth ER membrane joins with a primordial lipoprotein consisting of apolipoproteins, phospholipids, etc that are formed in the rough ER. Once formed, the pre-chylomicron is transported via a pre-chylomicron transport vesicle (PCTV) to the Golgi apparatus for further maturation.^54^ Eventually the mature chylomicron is exocytosed from the enterocyte into the lamina propria from where it is transported away via the lacteals to the lymphatic system due to the presence of open button-like junctions that enable entry between the lymphatic endothelial cells of lacteals.^5^ While long-chain fatty acids (≥16 carbon) and MAGs are mostly transported away from the intestine via the lymphatics,^55^ more polar medium chain fatty acids (6–12 carbons) are directly transported (bound to albumin) via the mesenteric blood capillaries to the portal vein and liver; where they are rapidly oxidized or incorporated into very low-density lipoproteins (VLDLs).

### Proteins

Ingested dietary proteins are partially digested in the stomach where protease pepsin cleaves the peptide bonds at aromatic AAs leading to the production of smaller peptides and AAs (the building blocks of proteins), which are essential for metabolic homeostasis. In the small intestine, pancreatic enzymes (trypsin, chymotrypsin, and carboxypeptidases) further hydrolyze dietary proteins into smaller length peptides (oligopeptides) and AAs. These serve as the major fuel supply for the small intestine and precursors for the synthesis of biomolecules including glutamine, nucleotides, nitric oxide (NO), and other regulatory proteins.^56^ In particular, glutamine is the intestine’s major energy substrate which has also been shown to enhance cell proliferation, regulate TJPs, and inhibit proinflammatory signaling.^57^

The majority of dietary AAs and peptides appear to be absorbed in the proximal jejunum and ileum.^58^ However, others have reported the colon as an absorption site but only responsible for 5% of the total intestinal absorption of AAs.^59^ The uptake of AAs across the AP is a complex process involving numerous transporter systems expressed on the brush border membrane (BBM) of human enterocytes, which may exhibit solute specificity and possibly operate as electroneutral or electrogenic transporters.^58^ Luminal peptides are further broken down into free AAs and smaller peptides by BBM peptidases. Peptides containing ≤4 AAs are mainly taken up in the proximal small intestine via BBM peptide transporter (PepT1) on the intestinal epithelial cells (IECs).^60^ In the enterocyte, cytoplasmic peptidase converts these peptides into AAs, which are then transported through BLP, although the transport of most amino acids requires Na-K-ATPase to maintain an efficient transcellular Na gradient (Figure 2). From the BLP, amino acids are transported to the portal circulation leading to the liver.^60^ Dysregulation of nutrient absorption and metabolism has been associated with various diseases such as inflammatory bowel disease, obesity, T2DM, and NAFLD/NASH.

## Macronutrients and metabolic dysfunction

### Carbohydrates and metabolic dysfunction

Many studies have now described the detrimental effects of excessive sugar consumption on the gut and liver, especially when ingested in the form of SSBs, which promote the development of metabolic diseases including obesity, NAFLD, T2DM, and cancer.^61^^,^^62^ A recent study has shown that chronic consumption of HFrD induces *de novo* lipogenesis and hepatosteatosis, which eventually progress to NASH, tumorigenesis, and hepatocellular carcinoma (HCC) in mice.^24^ When nonhuman primates were fed a calorically controlled HFrD, they exhibited increased biomarkers of liver damage, endotoxemia, and elevated hepatitis, concomitant with increased microbial presence in the liver.^63^

How dietary-derived sugars precisely drive gut and liver pathophysiology is not fully understood. While a fructose-rich diet has been recognized as the major catalyst by some,^64^ others suggest the major driver is simply excessive caloric consumption.^65^^,^^66^ In relation to these views, a six months-long randomized intervention with a fructose-sweetened beverage (FrSB) substantially increased fat storage in the liver and muscle and visceral fat, whereas other beverages (skim milk, diet soda, or water) failed to produce similar phenotypes in humans.^67^ Interestingly, a separate study reported that consumption of isocaloric quantities of both glucose and fructose resulted in similar weight gain; however, consumption of FrSB, but not a glucose drink, increased dyslipidaemia, visceral adiposity, and insulin resistance in humans with obesity,^26^ suggesting more deleterious effects of fructose when compared with glucose. Hieronimus, Medici, Bremer, Lee, Nunez, Sigala, Keim, Havel, and Stanhope^68^ asserted that a far more detrimental effect is observed in young adults when the two sugars are consumed together in the form of high fructose corn syrup (HFCS), echoing the work by Bray, Nielsen, and Popkin.^69^ Likewise, a human study involving lean female subjects demonstrated that four days of overfeeding with 50% sucrose drinks increased *de novo* lipogenesis by 300%.^70^ The observations that fructose was unable to increase weight gain over glucose^26^ and that leptin and ghrelin (gut-derived hormones that regulate appetite and metabolism) were not influenced by HFCS consumption,^71^ in part, formed the assumption that HFCS may not be a major cause of obesity.^72^ Considering that dietary fructose is usually consumed as sucrose or HFCS, it’s conceived that the metabolic effects associated with fructose could potentially be driven by caloric excess but not fructose per se.^2^

Although these studies have advanced our understanding of the antithetical role of fructose in metabolic diseases, it’s believed that the fructose exposure used in experiments usually exceeds 50% of the total physiological energy requirement.^43^ This may not reflect human consumption where, even in the 95^th^ percentile, fructose constitutes only 15% of total energy intake.^73^ Despite these findings, whether adverse metabolic effects are induced by fructose exclusively or result from excessive energy intake needs to be clearly defined. An interesting work by Taylor, Ramsamooj, Liang, Katti, Pozovskiy, Vasan, Hwang, Nahiyaan, Francoeur, Schatoff, Johnson, Shah, Dannenberg, Sebra, Dow, Cantley, Rhee, and Goncalves^74^ argued that small dose fructose increases intestinal cell survival and lipid absorption, suggesting a key role in promoting obesity. Taylor further demonstrated that fructose exposure in hypoxic and normoxic HCT116 cells increased hypoxia-inducible factor 1α, which transcriptionally regulates barrier-protective genes (e.g., TJPs), mucin secretion, and intracellular ATP levels.^75^ While this is a significant finding, it contrasts the views outlined herein, which in most cases fructose ingestion alone does not induce obesity. Clearly, this is an interesting concept that warrants further discussion.

### Lipids and metabolic dysfunction

Lipotoxicity emerged as a major contributing factor to pathophysiological changes ranging from excessive hepatic lipid deposition to advanced inflammation, fibrosis, organelle dysfunction, and eventually NASH. Previous studies reported that, in the liver, accumulation of highly active lipids such as ceramides, free fatty acids (FFAs), free cholesterol (FC), and DAGs hastens the development of NASH by modifying both the tissue biology and intracellular organelle function, mostly through ER stress and mitochondrial pathways.^76^

Ceramides are bioactive lipid metabolites, released from membrane sphingomyelin by sphingomyelinases, implicated in insulin resistance, inflammation, oxidative stress, and cell death.^77^ A *de novo* pathway of ceramide synthesis also occurs at the ER via the enzyme serine palmitoyltransferase 1 activation.^78^ Increased levels of ceramides have been linked to elevated proinflammatory cytokine levels, including interleukin (IL)-1 and IL-6, in both murine models and humans with NASH.^79^ Thus, ceramides interaction with tumor necrosis factor (TNF) is now considered as a plausible mechanistic process driving liver inflammation and NASH.^80^

The long-chain saturated FFAs palmitate (PA, C16:0) and stearate (C18:0) constitute a large portion of fatty acid in our diets and can be synthesized *de novo* from carbohydrates.^81^ Saturated FFAs have been implicated in toxic effects to hepatocytes through several mechanisms including TLRs and nuclear factor kappa β (NF-κB) activation, mixed-lineage kinase-3 (MLK3) and c-Jun N-terminal kinases (cJNK) signaling, activation of ER response, and engagement with Bcl-2 proteins.^82^ Notwithstanding their contributions to steatosis, some monounsaturated FFAs such as oleate (C18:1) and palmitoleate (C16:1) are thought to be less toxic to hepatocytes compared to other FFAs (e.g. PA). In fact, these lipids are thought to protect against cell death,^83^ supporting the views that excessive intracellular fat deposits may not usually lead to disease progression, and as such the effects of fat accumulation and lipotoxicity may not be synonymous.

Disruption in cholesterol transport and homeostasis can lead to the accumulation of FC in cells, an emerging aspect of hepatic lipotoxicity. Previous studies have linked dysregulation of cholesterol metabolism genes with elevated FC levels in NAFLD and further demonstrated that increased FC induced liver damage via c-Jun N-terminal protein kinase 1 (JNK-1) and TLR4-dependent mechanisms.^84^^,^^85^ Excess FC has also been associated with hepatic mitochondrial loading, which promotes steatohepatitis through a TNF-Fas-mediated mechanism.^86^ In steatohepatitis, Kupffer cells (a form of liver-resident macrophages) surround dead hepatocytes containing the cholesterol crystals to form crown-like structures. The Kupfer cells may then engulf the trapped lipids and transform into form cells furthering the development of NASH.^87^^,^^88^

## The gut microbiota and its function

The adult mammalian gut contains a complex symbiotic community of microorganism comprising of all three life forms—archaea, bacteria, and eukaryote. Besides gut bacteria, the crucial role of gut viruses and fungi in both health and disease has recently emerged.^89^^,^^90^ Although viruses are not considered as part of the microbiota, it’s believed the human gut virome may share similar trajectories with the gut bacteria, indicating their metabolic close interaction.^89^ Interestingly, severe COVID-19 infection was linked to lower beneficial commensal and higher opportunistic pathogens in the guts of human patients.^91^ Despite their known pathogenicity, it’s now appreciated that gut mycobiome (the fungal component of the microbiota) can engage in a commensal or mutualistic interaction with the host. Like bacteria, the mycobiome-host ecological bond can be modulated by diet^92^; and this may contribute to either gut health by promoting intestinal barrier function^93^ or metabolic dysfunctions such as obesity and liver diseases.^94^^,^^95^ Despite this renewed scientific interest, the inter-kingdom interaction of these microbial groups, and how this may affect gut and host metabolic function, is largely unknown.

The largest and most widely studied group of the gut microbiota is bacteria. The adult human gut contains enormously diverse colonies of at least 10^12^ bacteria. The composition and density of the gut bacteria differs along the length of the gastrointestinal (GI ) tract ranging from a narrowly diverse and lower density (expressed in colony forming unit [CFU], per milliliter) in the stomach and duodenum (10^1^–10^3^ CFU/mL) and jejunum (10^4^–10^7^ CFU/mL) to a wide variety and higher abundance (10^11^–10^12^ CFU/mL) in the large intestines.^96^^,^^97^ Such ecological pattern is dictated by several factors which facilitate distinct regiospecific phenotypes in the luminal tract. These include higher content of O_2_, primary bile acids (BAs), and nutrient availability at the proximal intestine and increased pH, mucin, secondary BAs, and endotoxin concentration toward the distal intestine.^98^ Acidic pH and primary BAs, coupled with short transit time in the small intestine provide an unstable environment for microbial colonization in contrast to the neutral pH and a much slower transit time in the large intestine, harboring the largest microbial community.^99^ Therefore, bacteria involved in fat and CHO metabolism (e.g. Enterococcus and Proteobacteria) predominantly populate the aerobic small intestine, while acidic-intolerant and fiber-metabolizing bacteria (e.g. Prevotella and Ruminococcus) are mainly found in the colon.^97^^,^^100^

The normal microbiota in the gut is broadly categorized into two major phyla: Firmicutes (including genera *Clostridium*, *Enterococcus*, *Lactobacillus*, and *Ruminococcus*) and Bacteroidetes (including genera *Bacteroides* and *Prevotella*).^101^ From Bacteroidetes, *Bacteroides* (associated with consumption of animal products) are inflammatory and are linked to metabolic diseases, whereas *Prevotella* (associated with consumption of a plant-based diet) are anti-inflammatory and otherwise protective. Most *Ruminococcus* and *Clostridium* species of Firmicutes are mainly associated with consumption of animal products and positively associated with diseases pathology. However, *Lactobacillus* species appear to exhibit disease-protective properties.^102^

The metabolic propensity of the gut bacteria is possibly influenced by their ability to produce gut metabolites, from dietary sources, which regulate various metabolic functions both in disease and health. For example, aerobic bacteria in the small intestine (including Bifidobacterium, Streptococcus, Propionibacterium, Stenotrophomonas, and Lactobacillus spp.) can breakdown linoleic acids to fatty acid derivatives with wide range of anti-inflammatory properties.^103^ In the large intestine, Anaerostipes, Bifidobacteria, Eubacterium, and Roseburia synthesize SCFAs (from undigestible CHO and protein sources) to maintain gut immune homeostasis and barrier integrity.^104^ SCFAs can upregulate the expression of barrier-protective genes (claudin, CLDN-1; mucin, Muc-2; and zonula occludens-1, ZO-1), thereby preventing the gut-liver axis translocation of endotoxins.^98^ However, SCFA have been reported to produce some obesogenic activities^105^ (discussed in subsequent sections). Likewise, certain bacteria including *Prevotella copri* and *Bacteroides vulgatus* may increase the production of branched-chain amino acids such as isoleucine and valine, which are implicated in insulin resistance and obesity, driving the development of NAFLD.^106^ In essence, increases in some bacteria colonies such as *Lactobacillus plantarum* have been shown to protect intestinal barrier integrity, thus preventing the gut content (e.g. bacterial toxins, protein, and partially digested fat) from entering into the blood stream.^107^ However, excessive caloric intake may modulate microbiota leading to dysbiosis and alter the synthesis of protective metabolites, triggering gut inflammatory responses.^108^

## The effects of macronutrients on the gut microbiota and gut metabolism

It’s now accepted that diet is a major factor that modulates the composition of gut microbiota.^109^ Dietary-induced changes in microbiota occur as different microbial species are genetically conditioned to utilize certain substrates, although bacteria generally favor carbohydrates as their primary energy source.^101^ Based on this, different macronutrients may prompt distinctive bacterial phenotypes in the gut. A landmark study that compared fecal microbiota of rural African children in Burkina Faso with European children revealed that Burkina Faso children consuming plant-based diets exhibited higher abundance of fecal *Bacteroidetes*, *Actinobatceria*, and *Prevotella*, whereas European children consuming a WD possessed a higher level of potentially pathogenic Firmicutes and gram-negative Proteobacteria, coupled with reduced SCFAs in the gut.^110^

Generally, excessive consumption of a high-caloric diet, typical of a WD style, is known to reduce gut microbial diversity, contributing to obesity and NASH, in contrast to a Mediterranean diet containing more vegetables, fruits, and red wine.^111^ This supports the early findings that high-caloric diet enhances microbial energy-harvesting capacity^112^ and that certain gut bacteria such as *Eubacterium rectale* and Clostridium coccoides have been correlated with obesity phenotypes (e.g., high BMI and serum triglycerides) in human female subjects.^113^ In a clinical study (NCT00414063) it was shown that a change in nutrient load (2,400 kcal/day vs 3,400 kcal/day) can alter gut microbiota. For example, excessive caloric intake was linked to increased Firmicutes-to-Bacteroidetes ratio in humans.^114^ Whereas, the opposite was observed in low-caloric diet, accompanied with decreased relative abundance of various bacterial taxa (e.g., *Clostridium ramosum*, Hungatella hathewayi, and Alistipi obesi) and reduced body weight in clinical investigations of human subjects with obesity,^115^^,^^116^ emphasizing the link between the microbiota and obesity.

Excessive ingestion of digestible carbohydrates in the form of starches and sugars (e.g., fructose and glucose) has been associated with dysbiosis-related disease pathologies. For example, consumption of the artificial sweetener saccharin resulted in gut dysbiosis by increasing relative abundance of *Bacteroides* while reducing anti-inflammatory *Lactobacillus reuteri*, an effect that is strongly linked to glucose intolerance.^117^ Congruently, mounting evidence demonstrates that consumption of HFrD increases inflammation-provoking bacterial load and alters bacterial composition.^118^^,^^119^^,^^120^ Two different studies in mice and rats have reported that high fructose ingestion increases the abundance of *Bacteroides fragilis* (a gram-negative bacteria), Coprococcus, Ruminococcus, and Clostridium (all gram-positive anaerobes).^120^^,^^121^ This is indicative of the detrimental effects of HFrD. A six-week intervention of sugar-sweetened drinks, containing 11% of total dietary sugar, increased disease-promoting Proteobacteria and Actinobacteria phyla, while caloric intake, body weight, and adiposity index remained unchanged in rats.^122^ In line with a previous observation,^63^ these data suggest that diet-induced changes in the gut microbiota possibly precede the ensuing metabolic phenotypes.

**Figure 3:**
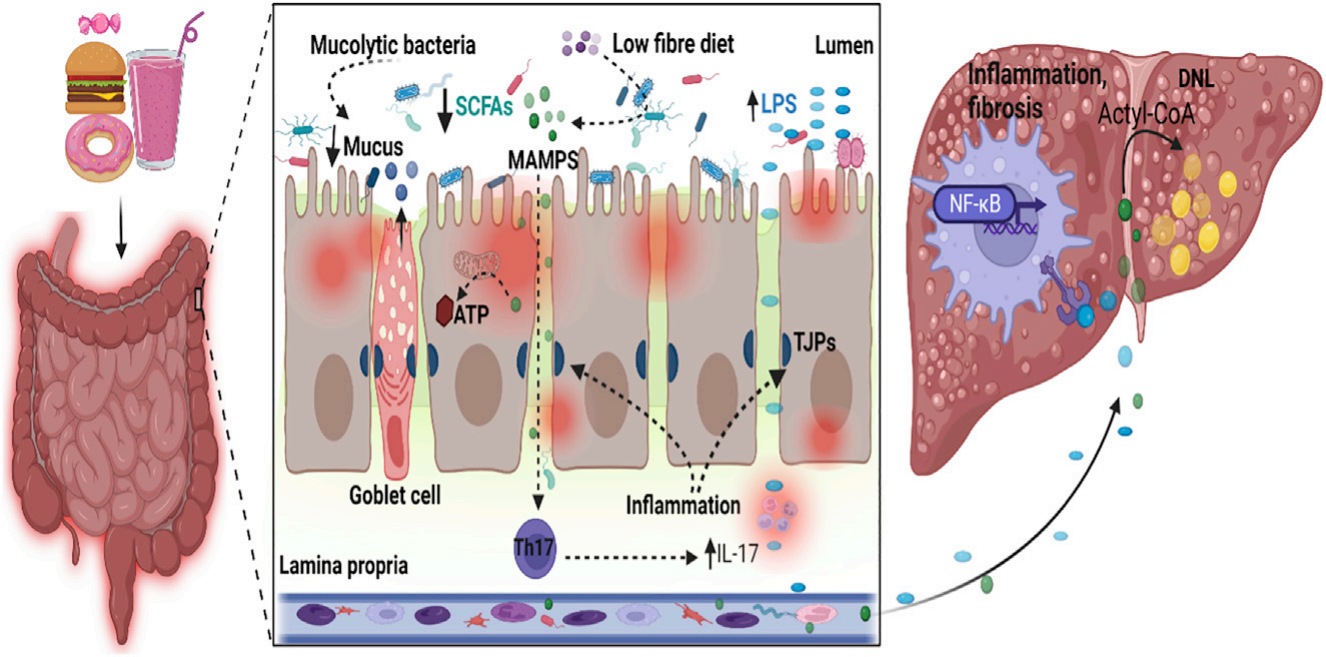
High-caloric diet (HCD) promotes gut permeability and NASH Highly processed food is rapidly absorbed in the small intestine depriving gut bacteria of essential nutrients, resulting in dysbiosis and increased production of bacterial endotoxins such as lipopolysaccharides (LPS). Mucolytic bacteria resort to mucus to obtain nutrients. This depletes the mucus layer and exposes enterocytes to luminal bacteria, triggering local inflammation. Because of the low fiber in HCD, very small quantities of SCFAs are produced, which reduces cellular ATP synthesis and the modulation of other immune functions. Coupled with inflammation, this reduces the production of mucin by the goblet cells. Chronic inflammation downregulates Ts (TJPs) leading to increased gut permeability. Gut-derived endotoxins leak out to the lamina propria. Microbe-associated molecular patterns (MAMPS) interact with immune cells (e.g. Th17) furthering the production of inflammatory cytokines such as interleukin 17 (IL-17). The gut endotoxins enter the portal vein and are transported to the liver. LPS binds to Toll-like receptor 4 (TLR4) and activates liver macrophages. The upregulation of nuclear factor-kB (NF-kB) transcription factor exacerbates hepatic inflammation and fibrosis. In the liver, SCFAs (acetate) feed into fatty acid synthesis pathways and are converted by acyl-synthetase to acyl-CoA. This subsequently generates triacylglycerols (TAGs) leading to increased *de novo* lipogenesis (DNL) and steatosis. Liver steatosis coupled with inflammation and/or fibrosis promotes the development of NASH. This figure was created with Biorender.com.

Western diet has been linked to gut dysbiosis, local inflammation, and intestinal hyperpermeability. An animal study demonstrated that mice fed high-fat and high-sugar diets exhibited reduced number of Bacteroidetes and increased the number of pathogenic Firmicutes and Mollicutes—a non-cell wall bacterial class.^123^ Genotypically, analysis of caecal microbiota from mice under WD influence revealed a relatively high abundance of genes implicated in gram-negative bacterial cell wall biosynthesis.^124^ Consistently, a separate investigation demonstrated that 4-weeks feeding of a WD significantly increased plasma endotoxin or LPS activity by 71%, whereas a prudent-style diet regimen reduced such activity by 38%.^125^ The relationship between a WD and dysbiosis is not well defined. However, it’s argued that WD is composed of ultra-processed acellular nutrients that are rapidly absorbed in the small intestine, thus depriving the colonic microbiota of essential nutrients for growth.^126^ Although the full impact of this on the microbiota may not have been determined, Desai, Seekatz, Koropatkin, Kamada, Hickey, Wolter, Pudlo, Kitamoto, Terrapon, Muller, Young, Henrissat, Wilmes, Stappenbeck, Núñez, and Martens^127^ explained that during dietary fiber deficiency, the microbiota resort to the gut mucosal barrier to obtain nutrients. This reduces the mucus layer and exposes the epithelial cells to lumenal pathogens such as *Citrobacter rodentium* (Figure 3). This may provoke local inflammatory responses leading to gut barrier deterioration and the development of NAFLD and NASH.

Like a WD, several investigations have also found that consumption of an HFD increased the number of mucin-degrading *Bacteroides, Faecalibacterium prausnitzii*, and promoted its excessive abundance in humans.^128^^,^^129^ Evidence from animal models has shown that an HFD noticeably reduces *Lactobacillus intestinalis* but induces the proliferation of certain gram-negative bacterial species such as *Enterobacteriales Clostridiales* and *Bacteroides*.^130^^,^^131^ In the study by Cani and colleagues, increased LPS was correlated with obesity and endotoxemia-induced inflammation in mice. Corresponding with recent data, HFD ingestion elevated the levels of some intestinal inflammatory markers (e.g., calprotectin and IL-6) in rats^132^, which in separate studies was shown to exacerbate dysbiosis-induced inflammation^133^ and compromise the gut barrier in mice.^134^

Dietary lipids have also been implicated in gut dysbiosis. A study by Caesar, Tremaroli, Kovatcheva-Datchary, Cani, and Bäckhed^135^ demonstrated that lard-derived lipids increased *Bacteroides* and *Bilophila*, resulting in exacerbated systemic TLR activation, white adipose tissue inflammation, and impaired insulin sensitivity. Whereas fish oil-derived lipids reversed these effects by increasing *Bifidobacterium*—known to reduce LPS levels—favoring a nonobese and anti-inflammatory bacterial composition.^135^ These findings demonstrate that although excessive caloric intake promotes gut dysregulation, different dietary lipids may elicit distinctive bacterial patterns and that metabolic inflammation via TLR signaling may be driven by a specific gut microbiota signature.

Although dietary proteins are generally correlated with positive microbial diversity, high consumption of animal-based proteins substantially increased the counts of pathogenic bile-tolerant anaerobes such as *Bacteroides*, *Alistipes,* and *Bilophila* in humans.^136^ This has been shown to activate bacterial enzymes capable of producing toxic metabolites (e.g., LPS), which consequently trigger inflammatory responses.^137^

On the contrary, plant-based protein extracts (from whey and peas) were reported to increase the healthy gut bacteria *Bifidobacterium* and *Lactobacillus*, concomitantly elevating the levels of SCFAs in the gut.^138^^,^^139^ The beneficial effects of plant-based diets are mainly attributed to their high fiber content, which allows more nutrients from undigestible fiber to reach the lower part of GI tract, thereby increasing bacterial population and diversity.^140^ Equally, natural sugars such as date fruits and nondigestible carbohydrates (including fiber and resistant starch) were shown to increase the abundance of gut commensal *Bifidobacteria* and *Lactobacilli* but reduced pathogenic *Bacteroides*.^141^^,^^142^ Collectively, these data show that consumption of a WD favors certain bacterial growth patterns, unlike that of plant-based diets. Nonetheless, dysbiosis alone may not induce NASH, but in concert with other factors such as inflammation and gut barrier deterioration could potentially hasten the development of NASH.

## Diet-induced gut dysbiosis and local inflammation: The host-microbiota interaction

As gut microbiota is constantly modulated by dietary changes, the host immunity must adapt to these alterations in order to maintain gut homeostasis. Commensal microbiota interacts with epithelial cells to arouse gut immune responses that protect the intestinal barrier. It should, however, be noted that overgrowth of commensal microbiota (due to their opportunistic nature) and excessive use of probiotics may also drive disease processes.^143^^,^^144^ To maintain physiological host-microbiota interaction, the host develops an adaptive selective process which boosts an immunological tolerance toward the symbiont community. It’s assumed that the microbiota possesses its own immune system, independent of the host, that recognizes bacterial and food antigens. This may signal the mucosal immune system, via luminal bacterial sampling by the intestinal microfold M cells, which function in immunosurveillance,^145^ to either initiate an inflammatory response or maintain homeostasis.^146^ Thus, the immune system possibly discriminates commensals from the pathogenic bacteria by inhibiting or activating innate immune signaling and regulatory T cells, respectively.^147^ In addition, luminal dietary metabolites can engage with endogenous ligands which may differentially regulate intestinal metabolic processes through various nuclear receptor signaling, thus fine-tuning intestinal immune response.^148^^,^^149^ Through this process, microbiota possibly modulates the gut immune response linked to metabolic diseases (e.g., obesity, T2DM, and NASH). For example, intestinal knockout of alkaline phosphatase, a gut barrier protector that deactivates LPS, was associated with weight gain in mice.^150^

The host reciprocally executes various defense mechanisms to monitor the intestinal signals such as the overgrowth of the gut bacteria.^146^ Mucus glycoproteins, secreted by goblet cells, are the primary physical defense barrier protecting the enterocytes from lumenal microbes.^151^ Enterocytes, the main cellular component of the gut barrier, are tightly linked by junction proteins such as occludins, claudin, E-catherins, and junctional adhesion molecules (JAMs), restricting the paracellular movement of intestinal content but selectively allowing active transcellular transport of nutrients.^152^

Several lines of defense prevent the permeation of microbes and their by-products across the gut barrier. Chemical defense mechanisms include several immune effectors such as antibacterial lectins, produced by intestinal Paneth cells to lyse bacterial cells, and protect the mucosal lining from bacterial invasion.^152^^,^^153^ In addition, immunoglobulin A (IgA) produced by mucosal plasma cells protects the gut barrier by neutralizing microbial pathogens. The stimulation of dendritic cells (DCs) in the Peyer’s patches by commensal microbiota products, to secrete cytokines (e.g. transforming growth factor β [TGF-β]), chemokines (e.g. CXCL13), and B-cell activating proteins, also leads to the production of IgA by plasma cells.^154^ Based on this evidence, dysregulation of commensal microbes may alter the gut barrier and elicit deleterious effects on the host.

Diet-induced gut dysbiosis (e.g., increase in mucin-degrading bacteria) has been implicated in dysregulated mucosal immune responses. This may lead to the activation of inflammatory pathways and local inflammation.^155^ Chronic mucosal inflammation, such as seen in the inflammatory bowel diseases (IBD), has been considered as a major contributing factor to the pathogenesis of NAFLD/NASH.^156^ Not surprisingly, the role of IBD in liver diseases and obesity has now been appreciated with an increasing number of IBD patients developing NAFLD (34%)^157^ or obesity (52%).^158^ Although NAFLD/NASH has usually been associated with obesity, emerging evidence has reported NAFLD/NASH in nonobese humans, with lean patients thought to have the worst phenotype.^159^^,^^160^ As mentioned earlier, HFrD-induced inflammation and barrier deterioration resulted in steatohepatitis in mice even in the absence of obesity.^24^ In this study, barrier restoration inhibited steatohepatitis highlighting the key role of dysbiosis-induced gut permeability in the development of NAFLD/NASH.

Several studies, in IBD models, have recognized that a major deleterious effect of gut inflammation is the deletion of goblet cells, which reduces mucin secretion and dysregulates IL-7 secretion (involved in defense mechanisms), furthering chronic inflammation and barrier deterioration.^161^^,^^162^ This is presumably initiated by the migration of monocytes into the intestinal lumen where they differentiate into macrophages and DCs, the latter of which increase the expression levels of TLR-2 and TLR4. Consequently, this promotes the production of inflammatory cytokines (such as IL-1, IL-6, TNF, IL-18, and IL-12) and the activation of CD40, an inflammatory marker, on DC surface.^163^ In the IECs, the activation of the TNF-TNFR2 signaling pathway upregulates the expression of myosin light-chain kinase (MLCK) resulting in disruption of TJPs molecular structure and increased gut permeability.^164^

Gut inflammation also drives the accumulation of unfolded protein in the lumen of the ER and activates intracellular signaling transduction pathways including unfolded protein response (UPR), thereby dysregulating some key genes including arginase 2 (*ARG2)*, X-box-binding protein 1 (*XBP1)*, and Orosomucoid-like 3 (*ORMDL3*) leading to ER stress.^165^ Taken together, these data, more or less, present a metabolic process in which chronic excessive dietary consumption induces gut dysbiosis, consequently prompting local inflammation. Persistent gut inflammation is now considered as a key contributing factor to increased gut permeability, which drives the pathogenesis of NAFLD and NASH.^166^ Notwithstanding, it’s believed that diet-induced gut dysbiosis takes place quite earlier^136^^,^^167^ than the overt changes, particularly in the liver. Therefore, it’s often difficult to draw the sequential relationship between the two events. As such, further time-controlled chronic animal studies are warranted.

## Died-induced dysbiosis and liver disease processes

### The leaky gut and the fatty liver—Nexus between the gut and the liver

The liver is anatomically located proximally to the gut with the blood supply connected by the portal vein. Thus, the liver is constantly exposed to intestine-derived components. Liver and gut communication is facilitated through bidirectional links involving the portal vein (from the gut to the liver) and biliary tract (from the liver to the gut).^168^ In healthy organisms, gut metabolites including nutrients and small quantities of bacterial products are absorbed via permselective transport and chiefly drained into the portal venous circulation to be carried to the liver, prior to entering the systemic circulation.

The liver normally develops tolerance against low exposure of gut-derived bacterial components, explained by the relatively low expression of TLRs and adaptor molecules such as MD-2 and MyD88 in liver tissues under physiological conditions.^169^ In conditions where the gut permeability is increased, however, more LPS (from gram-negative bacterial cell walls) is transported into the portal circulation by LPS-binding protein (LBP).^123^ The gram-positive bacterial cell wall also contains lipoteichoic acids (LTAs), which are amphiphilic molecules linked to a neutral glycolipid—a key immunostimulatory agent.^170^ Like LPS, LTA is also transported across the intestinal barrier by LBP. Upon reaching the liver, gut-derived metabolites interact with various hepatic cellular proteins such as TLRs, the results of which activate proinflammatory pathways driving the development of NASH.^171^ In addition to LPS and LTA, SCFAs have emerged as contributing factors in liver pathology. However, these metabolites are thought to be key features in normal healthy gut function.

## Can the gut’s good metabolites be the liver’s foes?

### LPS and hepatic TLRs activation

Intestinal bacterial metabolites are key substrates that maintain normal physiological functions of IECs.^172^ These bacterial metabolites include microbe-associated molecular patterns (MAMPS) such as LPS, flagellin, and peptidoglycan. In the gut, these bind to TLRs on the apical surface of the enterocytes, thereby stimulating the production of antibacterial proteins.^172^ This assumes a crucial role of TLRs in the intestinal host defense. Indeed, gut barrier protective responses were thought to be driven by TLRs’ recognition of MAMPS, which may maintain physiological distribution of TJPs and preserve epithelial function.^173^ A seminal study demonstrated that *Tlr2*^*−/−*^, *Tlr4*^*−/−*^, and *Myd88*^*−/−*^ mice present with defective IECs proliferation and gut barrier function, leading to substantial tissue damage and mucosal ulceration.^174^ In the liver, the extracellular leucine-rich-domain of TLRs recognize pathogen-associated molecular patterns (PAMPs), such as LPS, double-stranded RNA and DNA (dsNRA, dsDNA), and danger-associated molecular patterns (DAMPs), which activate downstream intracellular signaling cascade pathways contributing to the production of inflammatory agents.^175^

The mechanisms driving liver immune cell activation and inflammation are both complex and poorly defined. The ability of TLRs to recognize cell damage receptor signaling patterns is believed to initiate the early steps of liver inflammation. TLR4 specifically recognizes and binds gut-derived LPS (from gram-negative bacteria) via CD14 and MD-2 co-receptors, whereas TLR1 or TLR6 bind to lipoprotein moieties and peptidoglycan (from gram-positive bacteria).^176^ While bacterial flagellin is recognized by TLR 5, gut microbe-derived nucleic acids containing dsRNA and dsRNA are responsible for the activation of endosomal TLR3 and TLR9 pathways, respectively.^176^^,^^177^ Activation of TLR3 and TLR9 triggers intracellular signaling culminating in the activation of the inflammasome.^178^

Inflammasomes are multi-protein complexes found in the cytoplasm made up of one of various NLR and PYHIN proteins, mainly NLRP1, NLRP3, NLRC4, and AIM2. These play a key role in sensing PAMPs or DAMPs both endogenously and exogenously.^179^ It is suggested that PAMPS and DAMPS control the cleavage of the effector inflammatory cytokines (pro-IL-1β and pro-IL-18) into their mature bioactive forms. In particular, DAMPs have been shown to stimulate the generation of ROS, subsequently activating the NLRP3 complex.^180^^,^^181^ The binding of TLRs to Kupffer cells has been regarded the major route driving the production of these inflammatory agents (cytokines, chemokines, and ROS) in the liver.^182^ This triggers local hepatic inflammation and fibrosis, leading to NASH and hepatic carcinogenesis.^183^^,^^184^^,^^185^

### Short-chain fatty acids

SCFAs, with less than 6 carbon atoms, are the end products of bacterial fermentation of carbohydrates and proteins in the colon, albeit proteins are metabolized to produce branched-chain fatty acids such as iso-butyrate and iso-valerate.^186^ Acetate (C2), propionate (C3), and butyrate (C4) are the major dietary-derived bacterial metabolites and play a pivotal role in the gut immune regulation and barrier preservation. Acetate is involved in the secretion of mucin by goblet cells.^187^ Lack of acetate in germ-free mice (GFM) was associated with reduced Muc-2 O-glycans and compromised barrier integrity.^188^

Propionate and butyrate are involved in the regulation of intestinal immune physiology. As the main fuel source for colonocytes, butyrate modulates various actions including stimulation of mucus production, upregulation of TJPs, and maintenance of gut homeostasis.^189^ Its ability to upregulate the expression of TJPs such as claudin-1 and ZO-1 was found to reduce gut leakage and LPS translocation across the gut wall.^190^ This is possibly enhanced by the inhibition of NF-κB activation in lamina propria macrophages, thus dampening their responsiveness to commensal microbes.^191^

In the liver, however, SCFAs serve as precursors for lipogenesis, increasing the accumulation of triglycerides. Zhao, Jang, Liu, Uehara, Gilbert, Izzo, Zeng, Trefely, Fernandez, Carrer, Miller, Schug, Snyder, Gade, Titchenell, Rabinowitz, and Wellen^105^ reported that chronic high fructose ingestion increases the level of gut bacterial-derived acetate, which promotes DNL in the liver of mice. Previous works have identified the role of SCFAs in hepatic metabolism. Infusion of SCFAs into the cecum elevated the levels of the hepatic palmitate and cholesterol in mice.^192^ While propionate serves as a major precursor for hepatic gluconeogenesis, acetate is oxidized in the TCA cycle leading to the production of ketone bodies, cholesterol, and long-chain fatty acids. In addition, butyrate entering mitochondrial fatty acid oxidation is converted to acetyl-CoA joining the same pathways as acetate.^193^ Recent work from our laboratory has shown that fructose substrates through acetyl-CoA were converted to C16 and C18 saturated fatty acids by lipogenic enzymes (ACLY, ACC1, and FASN), promoting liver steatosis.^24^

## Therapeutic options

NASH is now the leading cause of liver transplantation in the United States, triggering a surge in the NASH drug market which is estimated to be worth $40 billion (USD) by 2025.^194^ As such, it’s now reported that over 30 clinical trials are being conducted for potential drug candidates to treat NASH.^13^ Despite this renewed interest, proper treatments for obesity-induced NASH are still limited. Lifestyle modification, through diet and exercise, and bariatric surgery are the only existing remedies available.^27^^,^^195^ However, the benefits of exercise are not well defined, and bariatric surgery carries potential health risks. Therefore, new therapeutic targets are urgently needed. Considering the significant role of the gut microbiota in the development of obesity-induced NASH, therapeutic inventions targeting the gut-liver axis are being keenly sought.^196^ As discussed early on, the gut-liver axis conveys the metabolic changes in gut microbiota to the liver, contributing to hepatic lipid dysregulation, inflammation, fibrosis, and cirrhosis. Each of these may be targeted for potential treatments.

### Diet restriction

Previous studies have explained the significance of caloric restriction to treating diet-related metabolic diseases such as obesity, T2DM and their associated complications including NAFLD, CVD, and cancer. While the rationale of reducing caloric intake presents a logical solution to reduce weight gains and hyperglycaemia, the feasibility of this approach may offer another challenge. Yoneyama, Crabbe, Ford, Murillo, and Finn^197^ observed that the addictive property of sweet taste in both caloric and noncaloric food sweeteners may encourage increased consumption preferences of aversive substances such as sugary beverages in mice models, limiting the choice to avoid. Recent work has linked sugar preference behavior to SGLT-1 signaling via vagus nerve excitability leading to neural activation in the caudal nucleus of the solitary tact region, mediated by the gut-brain axis.^198^ In addition, emerging evidence suggests alternative mechanisms of post-ingestion sugar sensing.^199^^,^^200^ These observations reveal that the drivers of increased consumption of high-sugary food are a mix of both the sweet taste and post-ingestive sugar-sensing mechanisms. Therefore, a dietary restriction approach may potentially be achieved through drug mechanisms targeting both these pathways. Of note, appetite-suppressant drugs including amphetamine-like molecules, liraglutide, and phentermine have also been made available.^201^ However, safety concerns have been raised.^202^ In addition to dietary restriction, pharmacological therapeutic interventions to treat obesity-induced NASH have also been explored.^203^^,^^204^

### Orlistat

Orlistat is lipase inhibitor that has been FDA-approved for the treatment of obesity due to its efficacy to prevent the breakdown of triglycerides into fatty acids and their absorption thereof in the gut.^205^ A double-blind randomized clinical study on orlistat showed an improved serum ALT, however, with no significant effects on NAFLD-related pathology.^206^

### IMM-124e

Derived from bovine colostrum (BC), IMM-124e, is an IgG-enhanced protein with potent therapeutic activities targeting the gut-liver axis. Previous work demonstrated that IMM-124e-containing BC reduces LPS influx from the gut, by neutralizing LPS molecules, thereby inhibiting enteropathogenic endotoxemia in animal models.^207^ BC contains insulin-like growth factor-1 (IGF-1) and thus was thought to regulate blood glucose. In a phase ½ clinical trial in biopsy-proven NASH patients, IMM-24e improved glycemic control and liver enzymes concomitant with increased glucagon-like peptide 1 (GLP-1), adiponectin, and T regulatory cells leading to improved NASH pathology.^208^

### Yaq-001

Yaq-001 is a nonabsorbable synthetic nanoparticle which adsorbs gut-derived bacterial toxins such as LPS, thus reducing gut microbial perturbations and the translocation of gut toxins to the liver. McNaughtan et al.^209^ revealed that Yaq-001 increases Firmicutes and decreases Bacteroides in rodents, leading to reduced LPS activities and restored immune function. Evaluation of the safety and tolerability of Yaq-001 in cirrhotic patients is currently being investigated in a first-in-human double-blinded multicentred trial (NCT 03202498).

### Fecal microbiota transplantation (FMT)

FMT has been extensively explored as a possible therapeutic option for the treatment of NAFLD/NASH and various metabolic diseases. Total FMT into diet-induced obese mice alleviated liver steatosis.^210^ In humans, FMT significantly improved the expression of hepatic functional genes, plasma metabolites, and liver steatohepatitis markers in a double-blind clinical study.^211^

### Farnesoid X receptor (FXR) agonists

FXR is a regulator of various metabolic pathways involved in the synthesis of bile acid and the regulation of lipoproteins.^212^ Its activation correlates with reduced hepatic gluconeogenesis, lipogenesis, and steatosis.^213^ The FXR ligand, obeticholic acid (OCA), resolved necro-inflammation and NASH in a phase III human clinical study (REGENERATE).^214^ However, in this study, OCA was associated with elevated LDL cholesterol levels in patients, and as such approval was rejected by the FDA in 2020. Several non-bile acid synthetic agonists (e.g., GS-9674, LMB763, and LJN452) developed to circumvent the disadvantages of OCA are currently undergoing phase II clinical studies.^204^ Interim data have, however, presented undesirable effects including serious adverse events and increased LDL cholesterol.^215^

### Ketohexokinase (KHK) inhibitors

KHK is a vital drug target for NASH due, in part, to its critical role in intracellular fructose metabolism. A newly developed KHK inhibitor (PF-06835919) reduced hyperinsulinemia and hypertriglyceridemia, coupled with reduced hepatocyte DNL and ChREBP in rats.^216^ A relevant concern, however, is the fructose-spillover effect, in which more fructose is shuttled to the liver and the colon, induced by intestinal KHK inhibition in animal studies.^217^ Interestingly, in a phase 2A study (NCT03256526), liver-specific KHK inhibition significantly reduced whole liver fat and inflammatory markers, suggesting a potential clinical benefit in the treatment of NASH.^218^

### Peroxisome proliferator-activated receptor (PPAR) agonists

PPARs are nuclear receptors with three isotypes (PPARα, PPARβ (also known as PPARδ), and PPARγ), but their functions target the same DNA segment to regulate metabolism and inflammation. While both PPARα and PPARδ are involved in hepatic glucose-lowering and anti-inflammatory effects, PPARα regulates fatty acids transports and β-oxidation.^219^ Activation of PPARα suppressed IL-1, IL-6, TNF, intercellular adhesion molecule 1 (ICAM1), and vascular cell adhesion molecule 1 (VCAM1) expression in the liver; resulting in increased plasma TG clearance in both *in vitro* and *in vivo* studies.^220^^,^^221^ A PPARα/δ receptors dual agonist (Elafibranor) was shown to alleviate liver injury and resolve NASH in a phase 2B trial (GOLDEN-505).^222^ However, it failed to meet the primary endpoints of NASH in phase 3 trials involving 2000 NASH patients and was consequently discontinued.^223^ Recently, a PPAR-α/γ dual agonist (Saroglitazar) exhibited promising data (improved liver injury, insulin resistance, and atherogenic dyslipidaemia) in Phase 2 trial (NCT03061721) in patients with NAFLD/NASH, with the hope to progress to phase 3.^224^

### ACC1/2 inhibitors

MK-4074 is a liver-specific ACC1/2 inhibitor that exhibited an effective reduction in hepatic TAG and lipogenesis by ≥ 30% after 1 month of treatment. Similar to OCA, MK-4074 was linked to elevated TG in both animals and humans; impeding its suitability.^225^ Considering the high rate of failure in therapeutic interventions as presented in these studies, others have postulated that new treatments for NASH, especially when driven by high-sugar dietary consumption, should specifically target inflammatory and ER stress pathways and intestinal barriers.^2^

### Interleukin 6 (IL-6)

IL-6 is produced and released by skeletal muscle following a nondamaging contraction, making muscle an endocrine organ.^226^ Wunderlich, Ströhle, Könner, Gruber, Tovar, Brönneke, Juntti-Berggren, Li, Van Rooijen, and Libert^227^ reported that blocking IL-6Rα signaling in hepatocytes promoted inflammatory cytokine secretion by Kupffer cells and increased systemic inflammation resulting in widespread insulin resistance. However, IL-6 transgenic mice exhibited improved leptin activities and ameliorated nutrient homeostasis.^228^ IL-6 also has inflammatory properties and has been associated with the development of liver cancer^229^ and colorectal cancer^230^, but its selective activation to target protective properties has proven complexity.

### Glycoprotein 130 (gp130) signaling molecules

A novel approach to block the adverse IL-6 signaling that triggers inflammation and obesity-related diseases is the administration of soluble gp130Fc (olamkicept), an IL-6 receptor that modulates the intracellular signaling transduction.^231^ Indeed, treatment with Olamkicept alleviated liver inflammation in a preclinical study in DIO mice.^232^ Furthermore, the activation of membrane-bound IL-6 receptor increased barrier protection and AMPK activation, thereby reducing liver steatosis and NASH.^24^^,^^233^^,^^234^ Based on this, a group from our laboratory engineered a designer cytokine, IC7Fc, that activates membrane-bound IL-6R signaling but not IL-6 *trans*-signaling activity.^235^ Made from the combination of two different cytokines (IL-6 and ciliary neurotrophic factor [CNTF]), IC7Fc modifies food intake and body weight and improves insulin resistance, thereby improving metabolic homeostasis in mice.^235^ ILC7Fc is an interesting candidate to test for therapeutic potential in NAFLD and NASH.

### Postbiotics

Postbiotics are probiotic-derived components including SCFAs, protein secretions (e.g., P40 and HM0539), bacteriocins, and endo- and exo-polysaccharides.^236^ Their immunomodulatory effects as well as bacterial inhibition activities enhance their protective role on intestinal barrier function. Gao, Li, Wan, Hu, Liu, Yang, Gong, Zeng, Wei, Yang, Zeng, He, Huang, and Cao^237^ have demonstrated that a *Lactobacillus rhamnosus* GG-derived postbiotic (HM0539) upregulated intestinal mucin (Muc-2) and TJPs (e.g. ZO-1), leading to increased protection against intestinal barrier injury induced by LPS and TNF treatments in mice. As mentioned earlier , however, translocation of these postbiotic, such as SCFAs, to the liver may promote NAFLD/NASH.

### High-density lipoproteins (HDLs)

HDLs are nano-sized carriers comprised of apolipoprotein A1 (ApoA1), phospholipids, and cholesterol. The biogenesis of HDL is mediated by ATP-binding cassette transporter A1 (ABCA1) in both the liver and the small intestine.^238^ The beneficial metabolic and anti-inflammatory effects of HDL have been appreciated in various conditions including cardiovascular protection,^239^ anti-infection,^240^ anti-hyperglycemic,^241^ and anti-inflammatory properties which have been reviewed by Murphy, Chin-Dusting, Sviridov, and Woollard.^242^ The ability of HDL to neutralize LPS furnishes a further potential therapeutic option. Emerging evidence has revealed that an increase in intestinal production of HDL can reduce gut-mediated liver injury by inhibiting gut-derived LPS-induced liver inflammation in both NASH and alcoholic steatohepatitis (ASH).^243^ As discussed earlier, intestinally derived LPS is transported to the liver by LBP, where its interaction with TLR4 receptor drives significant liver inflammation and fibrosis. The binding of HDL, in particular the intestinally produced HDL3, to LBP-LPS complex masks the LPS from TLR4+ liver macrophage detection, thereby inhibiting liver inflammation and fibrosis.^243^ Interestingly, intestinal HDL production was increased by an LXR agonist in this study. Therefore, intestinal-specific LXR agonists may provide therapeutic benefits for the treatment of NAFLD and NASH.

## Conclusion and future directions

Although macronutrients provide essential nutrients for body functions, excessive caloric intake contributes to energy imbalance and metabolic perturbations. Prolonged excessive consumption of HFD and SSBs is the major risk factor driving the growing incidences of metabolic diseases such as obesity, T2DM, NASH, and associated CVD and cancers. Despite advances in our understanding of the role of HFD and HFrD in liver pathology, the precise mechanisms by which specific macronutrients modulate metabolic pathways or whether one nutrient may have greater detrimental effects over the others needs further study. Gut microbiota has now emerged as a key player that modulates metabolism in both health and in disease pathology. Macronutrients, in particular HFD and HFrD, have profound effects on the gut microbiota linked to adverse outcomes on GI tract physiology, although the mechanisms involved are complex. Notwithstanding the complex mechanisms driving these disease processes, it’s plausible that gut dysbiosis, in most part, is a major contributor to the myriad diet-induced metabolic dysfunctions.

Gut dysbiosis is an activator of local inflammatory and ER stress pathways, and the released inflammatory cytokines exacerbate gut hyperpermeability. The development of hepatic DNL and inflammation is promoted by gut leakage in which LPS, bacterial acetate, and SCFAs are transported to the liver via portal vein to modulate hepatic metabolism and inflammation. This highlights the importance of the gut-liver axis in NASH. Diet-induced pathology affects a vast array of metabolic functions, and efforts to find suitable treatments have been met with significant challenges. New approaches that target gut and inflammation-related mechanisms have provided a new avenue for therapeutic interventions to treat NAFLD and NASH. Given the complexity of diet-induced obesity and NASH, more understanding into how specific dietary composition affects the disease process is needed. Therefore, further studies (under controlled conditions) to investigate the effects of each macronutrient in gut health and liver metabolic functions could provide vital insight.
